# Utilization of functional MRI in the diagnosis and management of cervical cancer

**DOI:** 10.3389/fonc.2022.1030967

**Published:** 2022-11-11

**Authors:** Hirsch Matani, Ankur K. Patel, Zachary D. Horne, Sushil Beriwal

**Affiliations:** Division of Radiation Oncology, Allegheny Health Network Cancer Institute, Pittsburgh, PA, United States

**Keywords:** cervical cancer, MRI, functional imaging, DCE- MRI, DWI-MRI

## Abstract

**Introduction:**

Imaging is integral part of cervical cancer management. Currently, MRI is used for staging, follow up and image guided adaptive brachytherapy. The ongoing IQ-EMBRACE sub-study is evaluating the use of MRI for functional imaging to aid in the assessment of hypoxia, metabolism, hemodynamics and tissue structure. This study reviews the current and potential future utilization of functional MRI imaging in diagnosis and management of cervical cancer.

**Methods:**

We searched PubMed for articles characterizing the uses of functional MRI (fMRI) for cervical cancer. The current literature regarding these techniques in diagnosis and outcomes for cervical cancer were then reviewed.

**Results:**

The most used fMRI techniques identified for use in cervical cancer include diffusion weighted imaging (DWI) and dynamic contrast enhancement (DCE). DCE-MRI indirectly reflects tumor perfusion and hypoxia. This has been utilized to either characterize a functional risk volume of tumor with low perfusion or to characterize at-risk tumor voxels by analyzing signal intensity both pre-treatment and during treatment. DCE imaging in these situations has been associated with local control and disease-free survival and may have predictive/prognostic significance, however this has not yet been clinically validated. DWI allows for creation of ADC maps, that assists with diagnosis of local malignancy or nodal disease with high sensitivity and specificity. DWI findings have also been correlated with local control and overall survival in patients with an incomplete response after definitive chemoradiotherapy and thus may assist with post-treatment follow up. Other imaging techniques used in some instances are MR-spectroscopy and perfusion weighted imaging. T2-weighted imaging remains the standard technique used for diagnosis and radiation treatment planning. In many instances, it is unclear what additional information functional-MRI techniques provide compared to standard MRI imaging.

**Conclusions:**

Functional MRI provides potential for improved diagnosis, prediction of treatment response and prognostication in cervical cancer. Specific sequences such as DCE, DWI and ADC need to be validated in a large prospective setting prior to widespread use. The ongoing IQ-EMBRACE study will provide important clinical information regarding these imaging modalities.

## Introduction

Among women, cervical carcinoma ranks fourth for both incidence and mortality worldwide. Within the United States in 2022, there are an estimated 14,100 cases and 4,280 deaths (1, 2). The most common histology is squamous cell carcinoma, accounting for 70-80% of all cervical cancers. Non-squamous histologies represent the minority of histologies, although are associated with worse prognosis (3). Prevalence of cervical cancer is strongly associated with socioeconomic status, in part due to differences in access to medical care and screening. Historically, staging based on the Federation of Gynecology and Obstetrics (FIGO) has been based upon clinical examination and limited imaging modalities including plain radiography, colposcopy, cystoscopy and proctoscopy, given the prevalence of these tumors within underdeveloped countries which often lack access to more advanced technologies. Without these technologies, defining the extent of primary tumor and the presence of pelvic and para-aortic lymph disease is difficult. Because of this, cross-sectional imaging is now included as an optional addition to assist with staging and prognostication, and assist with treatment (4). Imaging modalities commonly utilized in cervical cancer include magnetic resonance imaging (MRI) to assess the extent of local disease and define brachytherapy treatment volumes, and computed tomography (CT) or PET/CT to assess nodal status (5). Functional imaging is an even more novel approach being used to help define cervical cancer and its response to treatment, however its utility and implications on management have yet to be defined (6). This is a primary objective of the ongoing IQ-EMBRACE sub-study. In this study, MRI with T1, T2, diffusion and dynamic contrast-enhanced imaging will be obtained prior to treatment, along with diffusion-weighted and T2 imaging at the time of brachytherapy. Treatment will be delivered and patients will then be followed with this information to assess outcomes. As a novel imaging approach, we aim to review the current uses of functional imaging in the diagnosis and treatment cervical cancer.

## MRI techniques

Standard MRI is performed on the foundation of nuclear magnetic resonance. The main component of this is the spin of nuclei which is related to the nuclear makeup (7). A magnetic field is applied to these atoms resulting in the synchronous precession of protons resulting in “bulk magnetization” which can be represented as a single vector precessing about the magnetic field. This precessing magnetization is detected as well as subsequent relaxation of these protons allowing for both T1 and T2 weighted sequences (8). With improvements in technology, there has been an increasing interest in functional MRI sequences such as Dynamic-Contrast Enhanced (DCE) MRI, diffusion-weighted MRI (DWI), and perfusion weighted imaging. During Dynamic Contrast Enhanced Imaging a bolus of contrast agent is administered to the patient prior to imaging. Due to their low molecular weights, these agents are able to move across vessel walls in tumor and distribute into the extracellular space prior to being washed out. On T1-weighted MRI imaging, signal intensity increases are seen and so rapid image acquisition is performed to assess movement of the contrast agent in tumor cells. As tumor vasculature exhibits a large amount of permeability, the uptake is perfusion limited and relies mainly upon blood flow and vascular density versus vessel permeability. DCE is not able to provide a direct measurement of tumor hypoxia, however with its characteristics, can indirectly provide this information through assessing tumor physiology with low-molecular weight contrast agents (9). DWI is a standard imaging tool for disease processes such as stroke, and relies upon apparent diffusion coefficient maps (ADC). Important parameters of DWI imaging include “b-value” relating to the strength of the motion probing gradient, and resulting signal-to-noise ratio. This underlying mechanism allows for diffusion changes that can be mapped. Increased cellularity is a characteristic of malignancies and as a result, diffusion is impeded at imaging. Therefore, signal intensity of malignancies is higher than that of normal parenchyma (10). Perfusion measurements with MRI can also be obtained utilizing injection of an exogenous endovascular tracer. Arterial spin labeling is the idea of comparing the spin of inflowing blood water to stationary water in tissue. This tagging occurs looking at inversion of longitudinal magnetization. Images are obtained after a time delay to allow inflow after bolus reaches microvasculature and using signal differences, perfusion maps can be obtained (11). Lymphadenectomy is associated with high costs and so the addition of non-invasive methods for staging, specifically for detection of pelvic lymphadenopathy would be beneficial (12). The addition of MRI specifically for brachytherapy has also been associated with increased effectiveness of therapy and decreased costs by avoiding downstream cost of recurrence and management of toxicity from therapy (13). As a result, the costs of functional MRI techniques are expected to further provide information that can further improve diagnosis and therapies, and decrease costs. Along with costs, specific benefits of these MRI techniques are listed below.

## Current use of magnetic resonance imaging for cervical cancer

**Figure 1:**
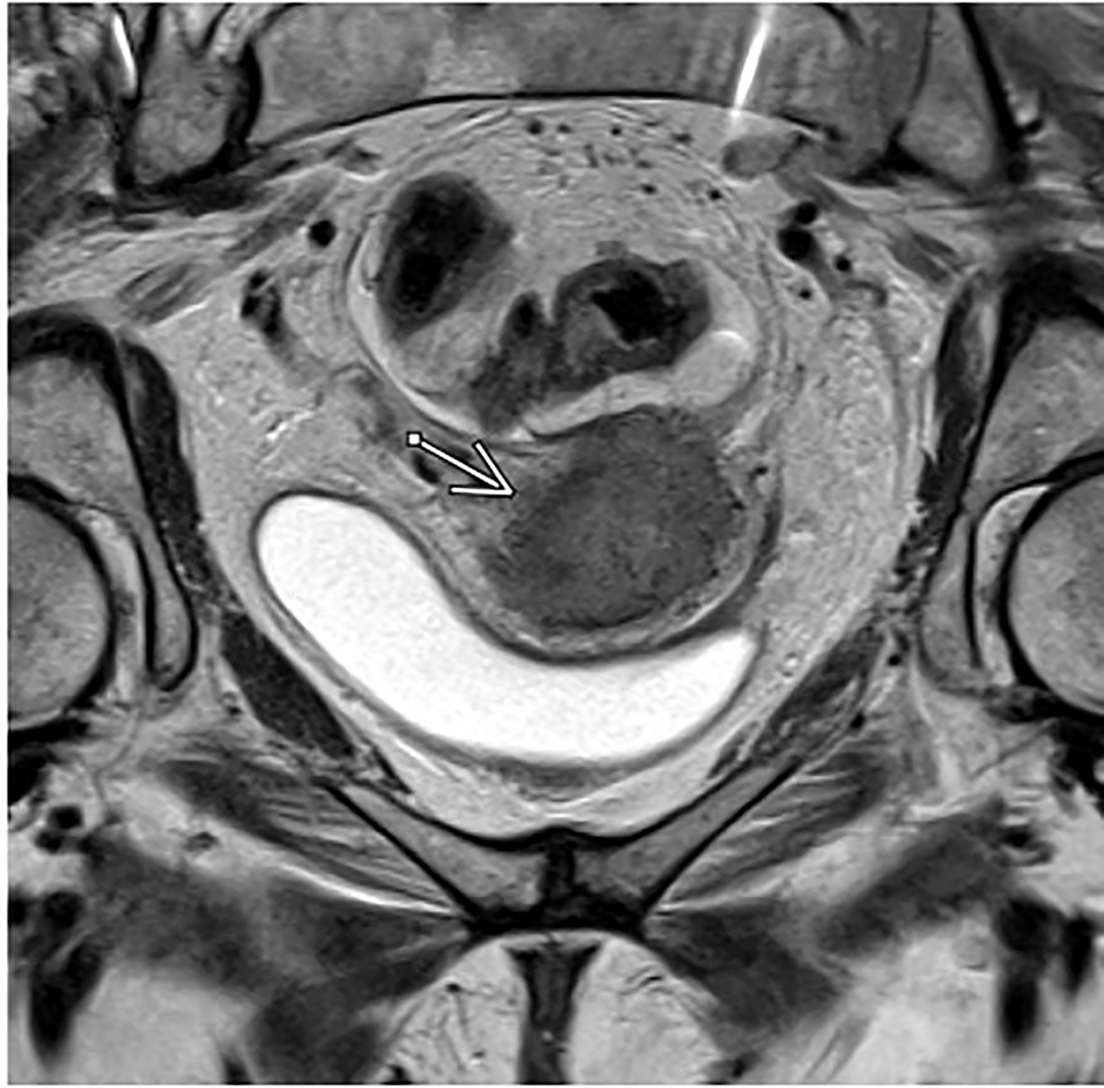
Axial T2-weighted MRI in a patient with FIGO IIIC1, pathologic T2 squamous cell carcinoma of the cervix.

Magnetic resonance imaging is an important component of staging for cervical cancer due to its superior soft tissue contrast resolution compared to CT. This characteristic makes MRI the preferred method for assessing the primary tumor and accurately assessing parametrial invasion and pelvic sidewall invasion with up to 95% accuracy or higher, ultimately allowing clinicians to determine the appropriate treatment modality (11, 14–17) (**Figure 1**). In addition, on the basis of the EMBRACE trials, MRI allows for image-guided adaptive brachytherapy (IGABT) with individualized target and organ at risk contouring, dose optimization and multiparametric dose prescription allowing for improved patient outcomes. In the Embrace cohort, 98% of patients could be treated with this method and overall local control was 92% across all stages, which was unprecendented (18). The mainstay of pelvic MRI to assess cervical tumors is T2 weighted imaging. Thin sections of 3-4mm are recommended and images should be acquired angled perpendicularly to the cervix (19).

## Dynamic contrast enhanced MRI

**Figure 2:**
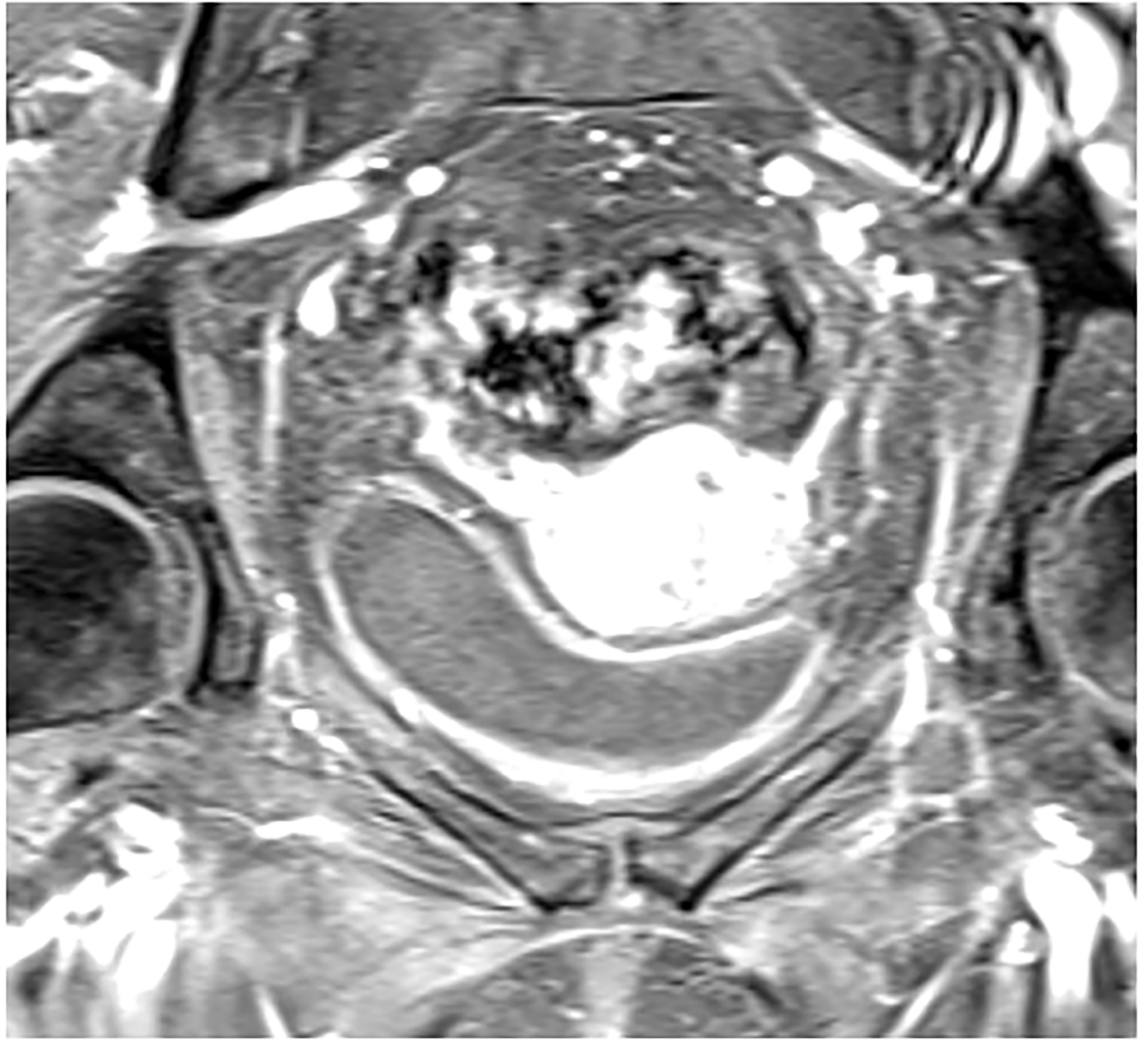
Post contrast dynamic contrast enhanced axial MRI showing increased signal intensity in a patient with FIGO IIIC1, pathologic T2 squamous cell carcinoma of the cervix.

Dynamic contrast enhanced (DCE) MRI is a widely accepted sequence of multi-parametric MRI for prostate and breast cancer imaging to assess suspicious findings on standard MRI and assist with treatment planning (20, 21). It is also the most studied functional imaging technique for locally advanced cervical cancer thus far and it is used to characterize tumor microvasculature, and therefore indirectly tumor perfusion status (22, 23). Hypoxia is a well-known feature of solid tumors and its presence is known to be associated with increased aggression of the tumor, increased risk of local invasion, metastasis and treatment failure. It is also known to influence the outcome of treatment with chemotherapy or radiotherapy, even in the case of microscopic tumor involvement (24). This is not surprising, as tumor hypoxia in cervical cancer specifically is an adverse risk factor and associated with poor outcomes, regardless of treatment modality (9). On this basis, DCE has been studied to predict for recurrence and survival outcomes in cervical cancer. (**Figure 2**) depicts tumor imaging on DCE. The largest study aimed to characterize a high risk “functional risk volume” of tumor voxels with low DCE signal intensity on MRI prior to and during radiation therapy. Those voxels included in the functional risk volume had a DCE signal intensity of <2.1 compared with pre-contrast imaging. At 6 years follow up, primary tumor control (p=0.003) and disease-specific survival (p=1.9 x 10^-4^) were significantly decreased in patients with a higher functional risk volume, and this parameter was superior to anatomic tumor volume as predictive and prognostic tool. The authors also found that high perfusion prior to treatment initiation or improved perfusion throughout treatment was associated with improved outcomes, which was hypothesized to reflect re-oxygenation and may have potential for monitoring response to therapy (25, 26). Validation of using DCE thresholds for characterizing voxels at risk for treatment failure has been performed, with findings similar to the prior study. Optimal signal intensity thresholds for differentiation of local recurrence and control ranged from 2.1-2.2 and for death and survival ranged from 1.8-2.2. A universal threshold based upon these values of 1.9 was identified for prediction of outcomes either prior to treatment or during treatment and this value remained significant for prediction of early treatment failures (27). The Mayr group has also published data regarding the use of DCE imaging at 2-2.5 weeks into radiation therapy to predict the risk of recurrence and death using a signal intensity cutoff of the lower 10^th^ percentile. Signal intensity was an independent predictor of recurrence and death and was significantly better than standard clinical prognostic factors in predicting outcomes (28). At least two studies, however have shown no correlation between DCE values and outcomes (29, 30). One study showed earlier onset of DCE enhancement to be associated with higher clinical stages, but not disease-free or overall survival. The other aimed to determine response to therapy by analyzing changes in tumor size and volume, for which pre-treatment DCE values did not correlate. One reason for this difference could be a lack of statistical power in the first study, which only had 12 patients evaluated and a short mean follow up of 11 years. Since DCE values are indicative of the tumor microenvironment and vasculature, they may not directly correlate with anatomic tumor size or volume, which is a measure of the total number of cells within a tumor. There is a variety of different techniques that have been used to evaluate DCE, but given that the bulk of data is positive, DCE-MRI has potential to be used as an important predictive or prognostic factor in management of cervical cancer, and to help determine who may benefit from escalation of therapy.

## Diffusion weighted imaging

Diffusion weighted imaging (DWI) is a method of signal contrast generation based upon differences in diffusion, also known as Brownian motion. Different tissues have a characteristic cellular architecture and pathologic processes can affect the water distribution within compartments, allowing for new anatomic information that can be gathered with conventional MRI sequences and is quantified by an apparent diffusion coefficient (ADC) map (31). Its current uses are mainly for diagnosis of central nervous system pathologies such as pediatric brain development, ischemic injury and white matter disease, although it has been used for diagnosis of malignancies throughout the body, as malignancies have a lower ADC compared with normal tissues (32). This feature is why diffusion-weighted MRI based imaging has been studied in the diagnosis of cervical and other gynecologic malignancies with some success, based on its ability to separate normal tissue from carcinomatous tissue (33). Particularly in the diagnosis of cervical cancer, a low ADC has been useful to help separate characterize the primary tumor as well as metastatic versus benign pelvic lymph nodes (34–37). Although the ADC values vary per study, using a value between 1.05 x 10^-3^ mm^2^s and 1.14 x 10^-3^ mm^2^/s as a cutoff yielded a sensitivity, specificity and accuracy as high as 95.83%, 94.55% and 94.94% (33). Another potential use of DWI is to characterize tumor histology. In one study, the ADC value was noted to be significantly lower for squamous cell carcinoma that for adenocarcinoma, however there was overlap between the two values and so currently it cannot be used to precisely separate the two (38). DWI has also been used to help characterize or predict treatment response. In one study out of Japan, for 9 patients ADC was correlated with response to chemotherapy and/or radiation therapy with an increase in mean ADC value of the lesion after treatment associated with adequate response (35). For squamous cell carcinoma specifically, the 90^th^ percentile of ADC values was lower in patients who responded to therapy compared to those who did not respond (39).

With all of these potential uses, DWI could play a significant role in the management of cervical malignancies through accurate diagnosis. This could help, particularly with patients who have equivocal post-treatment findings or lymph nodes after therapy. It is known that patients with adenocarcinoma have a worse prognosis with respect to progression free survival and local recurrence (40). If we are able to more accurately assess which patients with squamous cell carcinoma may be resistant to therapy or assist with histologic determination using DWI, we can potentially improve therapy to provide these patients with the best chance of controlling their disease.

A recent retrospective review of patients with medically inoperable stage I endometrial cancer treated with definitive radiation therapy found that the addition of DWI to standard post-contrast MRI to assess response to treatment increased reader confidence and the authors concluded that this sequence should be included as a standard addition (41). Another retrospective review out of MD Anderson Cancer Center analyzed patients with cervical cancer treated with definitive chemoradiation who had an MRI scan with DWI performed at baseline. They found that those with a higher mean pre-treatment ADC had improved disease-free survival with a trend toward improved overall survival and local recurrence. These outcomes remain similar to those from other institutions (42, 43).

**Figure 3:**
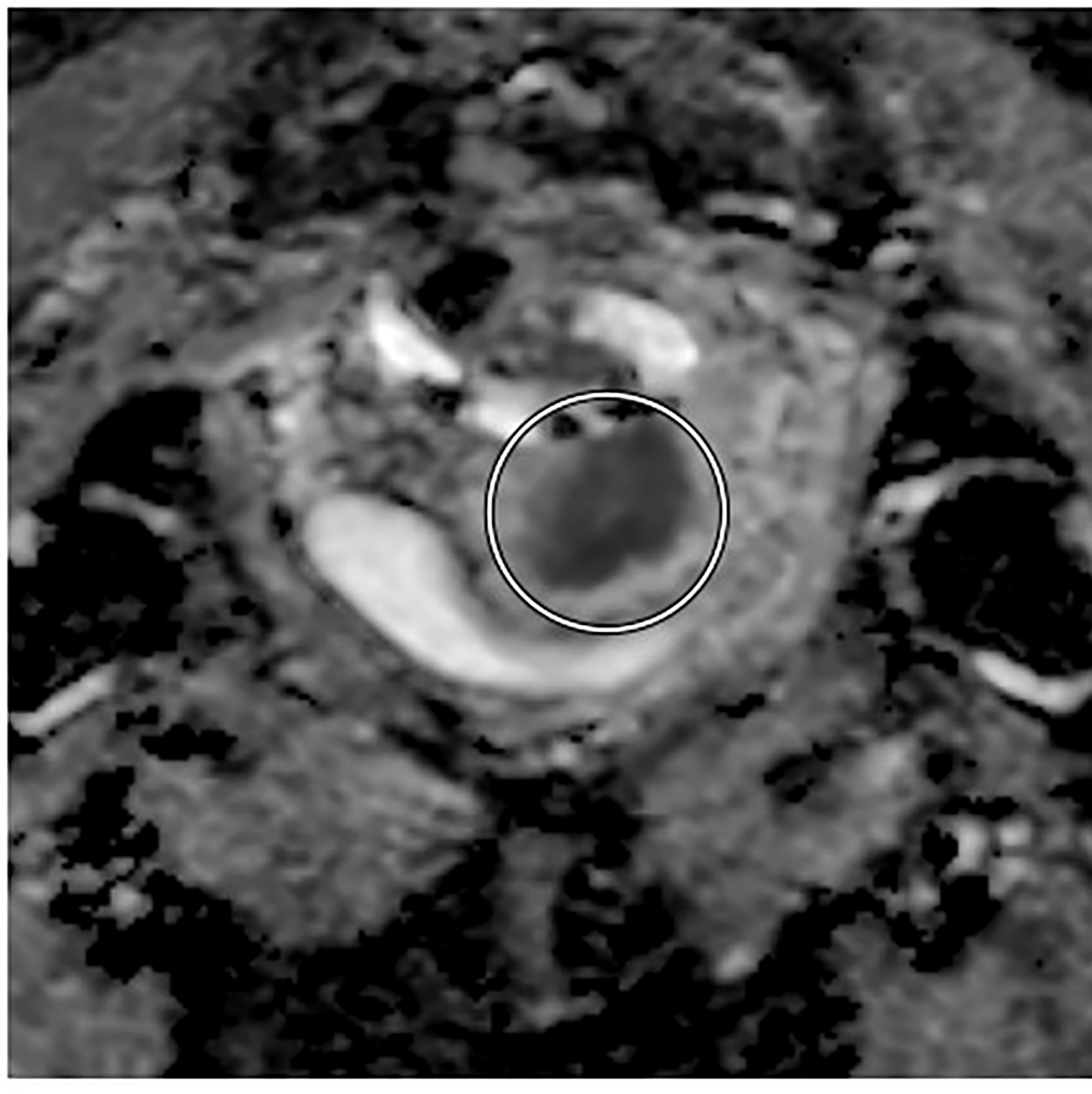
Axial diffusion-weighted MRI showing pre-treatment decreased tumor signal intensity in a patient with FIGO IIIC1, pathologic T2 squamous carcinoma of the cervix.

For post-treatment follow up, the addition of DWI imaging has been shown to increase the early detection of patients who have a PET incomplete response after completion of definitive chemoradiotherapy, in a large retrospective review by Kalash et al. (**Figure 3**). Of 27 patients with a PET incomplete response who had DWI imaging, 11 were interpreted as DWI positive, with a median ADC of 0.973 x 10^-3^ mm^2^/s. Of those 11 patients, 81.8% experienced a histologically confirmed local recurrence at mean interval of 4.1 months and of the 16 patients with negative response, only 12.5% experienced a local recurrence. In addition, a positive result on DWI was associated with significantly decreased local control at 2 years (92% vs. 20%, p<0.005) and decreased overall survival (83% vs. 36%, p=0.049). Overall the positive predictive value was 81.8% and negative predictive value was 87.5% (44).

Regarding its use for radiation treatment planning and contouring, only one prospective study performed in India has reported on the use of DWI in conjunction with T2 weighted MRI for target delineation for delivering MRI-guided adaptive brachytherapy and reported that per the GEC-ESTRO contouring guidelines, although the DWI based plan resulted in improved coverage of the high-risk CTV, doses to organs at risk were not significantly increased and DWI is recommended only as a supplement to standard T2-weighted imaging in the delivery of radiation therapy (45).

## Perfusion-weighted imaging

Perfusion is defined as the steady-state delivery of blood to an element of tissue and specific MRI techniques have been developed to measure this non-invasively. The two major MRI sequences to look at perfusion include use of DCE-MRI as briefly described above and dynamic susceptibility contrast-enhanced MRI. DCE MRI is obtained after injecting a contrast agent, often gadolinium-based and obtaining T1-weighted sequences to assess enhancement and other pharmacokinetic features. The most frequently used metric is ktrans which can reflect blood flow or vessel permeability depending on anatomic characteristics. Dynamic susceptibility contrast-enhanced (DSC) sequences also require injection of a contrast agent, but rely on T2-weighted sequences leading to MRI hypointensity. Cerebral blood volume and cerebral blood flow maps can then be derived from this information (46). Perfusion features of DCE have been described above, but briefly, DCE-MRI has been used as a predictive and prognostic tool. Low DCE-perfusion values are associated with poor response to therapy, local control and disease-free survival. They have also been shown in some small studies to predict response to therapy and early recurrences (25–27). Only one study has been recently published specifically looking at the role of DSC-MRI in management of cervical cancer. DSC-MRI values pre- and post-concurrent chemoradiation were examined and it was found that perfusion fraction of the tumor prior to concurrent chemoradiotherapy was higher in patients who had a partial response to therapy compared to those who had stable disease or progression (47).

## MRI-spectroscopy

MRI-spectroscopy is a functional MRI technique that assesses the presence of small mobile molecules *in vivo* at the commonly used MRI strengths of 1.5T and 3T. In normal tissues, these molecules are choline, creatinine and N-acetyl-aspartate, whereas pathologic conditions including malignancies may increase the presence and detection of other molecules such as lactate or alanine (48). It is commonly used in the diagnosis of both benign and malignant central nervous system pathologies as well as malignancies throughout the body including prostate, colon, breast and cervical cancers (49, 50). Only a handful of published data is found regarding this technique in the diagnosis and management of cervical cancers. The most data, is regarding the analysis of lipid levels using *in-vivo* and ex-vivo samples to diagnose cervical cancer. Use of MR-spectroscopy to detect lipid levels, in particular in-phase triglyceride (CH)-2 *in vivo* and triglyceride (CH)-2 and (CH)-3 ex-vivo as able to predict the presence of cervical cancer compared to control subjects with an *in-vivo* sensitivity and specificity of 77.4% and 93.8%. Ex-vivo sensitivity was 100% and specificity was 69%.55 Analysis of lipid levels for diagnosis has been corroborated by multiple groups (51–53). Using a 3T MRI to evaluate the presence of choline was unfortunately however not significantly different between different lesion types or in benign versus malignant disease (54).

To assess disease response, only a few studies have investigated variations in metabolite peaks during or after completion of treatment. Resolution of choline peak is correlated with tumor regression after radiation therapy, however its predictive value and clinical utility remain uncertain (55). This technique has also been used to identify increased choline signal along with T2-weighted imaging and ADC to guide adaptive radiotherapy, but outcomes thus far are unknown (56).

## Future directions/conclusions

DCE and DWI are imaging techniques that have been useful to assist with diagnosis of cervical cancer as well as for its predictive/prognostic value. Future studies will be aimed at providing validation of their capabilities and identifying the specific clinical settings in which they provide the most additional utility. Currently, this appears to be their role in post-treatment follow up and management. Prospective data with larger sample sizes are needed and will be provided in the IQ-Embrace Cohort. MRI spectroscopy and other functional MRI sequences should be further investigated along with DCE and DWI to assess clinical uses and help improve care for cervical cancer patients.

## Author contributions

HM contributed to the study design, data acquisition and writing the first draft of the manuscript. AP contributed to study design and data acquisition. ZH contributed to study design and data acquisition. SB contributed to conception of the study, study design and data acquisition. All authors contributed to manuscript revision, read and approved the submitted version.

## Conflict of interest

The authors declare that the research was conducted in the absence of any commercial or financial relationships that could be construed as a potential conflict of interest.

## Publisher’s note

All claims expressed in this article are solely those of the authors and do not necessarily represent those of their affiliated organizations, or those of the publisher, the editors and the reviewers. Any product that may be evaluated in this article, or claim that may be made by its manufacturer, is not guaranteed or endorsed by the publisher.
